# Endothelial cells regulate mesangial cells through the Dll4/Notch3 axis to participate in glomerular injury in lupus nephritis

**DOI:** 10.3389/fimmu.2026.1720756

**Published:** 2026-03-13

**Authors:** Haixia Guo, Yueying Mao, Yongjun Wang, Bin Yu, Zhifeng Gu, Chun Yao, Zhanyun Da

**Affiliations:** 1 Department of Rheumatology, Affiliated Hospital of Nantong University, Medical School of Nantong University, Nantong, Jiangsu, China; 2 Department of Endocrinology and Rheumatology, Tinghu District People’s Hospital, Yancheng, Jiangsu, China; 3 Key Laboratory of Neuroregeneration of Jiangsu and Ministry of Education, Co-innovation Center of Neuroregeneration, Nantong University, Nantong, Jiangsu, China

**Keywords:** Dll4/Notch3, endothelial cells, lupus nephritis, mesangial cells, tarextumab

## Abstract

**Introduction:**

Increasing evidence suggests that microvascular dysregulation mediates mesangial cell (MC) alteration in the pathogenesis of LN (Lupus nephritis). However, the heterogeneity of endothelial cells (ECs) in LN and their regulatory mechanisms on MCs have not been thoroughly investigated.

**Objective:**

To elucidate the regulatory role and mechanism of ECs in the development and progression of LN.

**Methods:**

We analyzed EC heterogeneity and cell-cell interactions in the kidneys of LN mice using single-nucleus RNA sequencing (snRNA-seq). *In vitro*, MCs were treated with Dll4 protein or the Notch3 inhibitor (Tarextumab) to detect the effects of Dll4/Notch3 axis on MCs. Then, in an EC and MC co-culture system, we interfered Dll4 in ECs or added Tarextumab in MCs to confirm that ECs can secret Dll4 to regulate MCs by Notch3 pathway after LPS stimulation. ELISA and immunohistochemical staining were performed on clinical blood samples and renal biopsies, respectively, to compare Dll4 expression levels between systemic lupus erythematosus (SLE) patients without LN (non-renal SLE) and those with LN.

**Results:**

We found that the number of ECs was increased during LN process and ECs exhibited cellular heterogeneity, among which EC subcluster EC-1 was identified as a potential regulator of MCs, possibly through the Dll4/Notch3 axis. *In vitro* experiments showed that Dll4 can promote the proliferation and migration of MC through Notch3. Interfering Dll4 in ECs or administering a Notch3 inhibitor (Tarextumab) in MC can significantly inhibit the effects on MC. Clinically, we analyzed peripheral blood and kidney tissues from non-renal SLE and LN patients and confirmed that Dll4 expression correlated with the disease activity of LN.

**Conclusions:**

Our findings suggest that abnormal EC activation may be associated with LN progression, potentially through promoting MC migration and proliferation via the Dll4/Notch3 axis. This study reveals a candidate molecular axis in the progression of LN and provides preliminary preclinical insights for future investigation into therapeutic strategies in LN.

## Introduction

The etiology of systemic lupus erythematosus (SLE) is complex and may result from the interplay between genetics, environment, hormones and immune regulation, including infections and dysbiosis (1–4). Lupus nephritis (LN) occurs in 40-60% of patients with SLE. LN is a common complication of SLE and is a determinant of overall morbidity and mortality (5–9). LN pathogenesis involves cell proliferation and autoantibody deposition in the glomerulus, leading to increased inflammatory response, disruption, and fibrosis in the kidneys (10, 11).

Recent studies have shown that the resident cells in the kidney, including mesangial cells (MCs), podocytes, and endothelial cells (ECs), may participate in inflammation and immune response in the glomerulus (12). MCs account for approximately one-third of the total cells in the glomerulus. They play critical roles in the development of the glomerulus and the maintenance of the capillary network structure in the normal adult glomerulus (13). MCs express a large number of antigens, which can bind to autoantibodies and are targets for damage in LN (14). They are also early responders to the deposition of immune complexes in the glomerulus and contribute to inflammation and fibrosis in LN (15). Previous studies by our group showed that MCs proliferate significantly with the progression of the disease in LN mice and revealed that macrophages regulate MCs through the CXCL12/DPP4 axis in LN pathogenesis (16).

The glomerular ECs, a component of the glomerular filtration barrier (GFB), line the glomerular capillary lumen (17). The integrity of ECs is essential for endothelial function and vascular health and is maintained through a dynamic balance between endothelial damage and repair (18). Studies have shown that the renal vascular damage are closely associated with clinical disease activity and renal outcomes (19, 20). The pathological alterations of ECs in LN are obvious (21). ECs can trigger inflammatory responses under appropriate pro-inflammatory stimulation. In the glomerulus, MCs are in direct contact with ECs (22), and communicate with ECs to maintain the integrity and normal function of the glomerular basement membrane (15). However, the role of activated ECs in communicating with MCs in LN remains incompletely understood. Recent research has revealed that vascular ECs exhibit heterogeneity in different tissues, and this heterogeneity plays a crucial role in various disease models (23, 24). Here, we analyze the ECs in the renal glomeruli of LN mice by single-nucleus RNA sequencing (snRNA-seq), to explore the changes in ECs and their subgroups at different LN stages, investigate the potential regulatory role of ECs on MCs, and explore intervention methods in the early stage of LN disease.

## Materials and methods

### Clinical study participants

Patients aged 30–55 years and diagnosed with non-renal SLE or LN, specifically those with type III or type IV LN, were recruited from the outpatient and inpatient services of the Rheumatic Immunity Department at the Affiliated Hospital of Nantong University. The primary pathological feature observed in these patients was MC proliferation. Plasma samples were collected from 55 participants, including healthy Chinese volunteers and patients newly diagnosed with SLE or LN. Clinically active SLE was defined by an SLE Disease Activity Index (SLEDAI)-2 K score greater than 6, whereas low disease activity was defined as an SLEDAI-2 K score of 4 or lower (25). During the same period, 15 plasma samples were obtained from age- and sex-matched healthy Chinese volunteers who served as the control group (Supplementary Tables. S1 and S2). Additionally, renal biopsy tissues from 3 LN patients with LN were screened, with three relatively healthy normal tissues from surgically resected specimens of patients with renal cancer used as controls. The clinical aspects of this study adhered to the ethical guidelines of the 1975 Declaration of Helsinki and all patients included gave written informed consent, and the research protocols were approved by the Institutional Review Board of the First Affiliated Hospital of Nantong University (approval number: 2025-L062).

### Clinical analyses

Levels of Dll4 in blood samples were measured by ELISA and the results were used in the basic clinical data analysis for the non-renal SLE and LN cases, while SLEDAI scores were used to assess SLE severity as described previously (**Supplementary Tables S1, S2**). The levels of Dll4 expression in human renal biopsy tissues were detected by Immunofluorescence staining.

### Animals

The MRL/MpJ-Faslpr/J (MRL/lpr) mouse is a classical model of spontaneous SLE and LN (26, 27). Female MRL/lpr mice at 6 and 10 weeks of age were obtained from Shanghai SLAC Laboratory Animal Company. All animals were maintained and used in accordance with the guidelines of the Institutional Animal Care of Nantong University. Both groups (n = 5 each) were fed ad libitum. Mice at 6 weeks of age served as the early-disease time point group, representing the initial stages of LN development. Mice at 10 weeks of age with established LN represented the progressive stage of the disease. Mice were euthanized by intraperitoneal injection of sodium pentobarbital (150 mg/kg body weight), followed by cervical dislocation. All procedures were approved by the Institutional Animal Ethics Committee of Nantong University (approval number: P20250225-013) and performed according to the guidelines of the Animal Welfare and Ethics of the Institutional Animal Care and Use Committee.

### The snRNA-seq analyses

All of the snRNA-seq analyses were performed by Guangzhou Gene Denovo Honour Biotechnology Co., Ltd. (https://www.omicsmart.com/home.html#), based on our previous snRNA-seq data (16). Based on the published data of mouse glomerular single-cell sequencing in recent years and the classification annotation of cells by classical marker genes, nine cell types were identified and isolated (28). To further investigate EC heterogeneity in LN progression, we performed unsupervised clustering on ECs and identified five transcriptionally distinct subclusters (EC-0 to EC-4). We then identified the top marker genes specifically expressed in each EC subcluster (see Supplementary Table. S5). GO enrichment analyses for the Differentially Expressed Genes (DEGs) were then performed using the Gene Ontology database (www.geneontology.Org) (29). We analyzed the expression abundance of ligand–receptor interactions between EC subpopulations and MCs by CellphoneDB, currently the most widely used cell communication analysis software available (30). Among them, only receptors and ligands that were expressed at levels greater than 10% were considered for the analysis (31).

### Histology and immunostainings

For immunohistochemistry (frozen sections), kidney tissues were collected and fixed in 4% paraformaldehyde for 24 h. After cane sugar dehydration, the kidney tissues were embedded in OCT glue and cut into 5-μm sections. Mouse glomerular tissue was labeled with CD31 (1:50, ab7388, Abcam, Cambridge, UK), Pi16 (1:100, PAQ943Mu01, Cloud-Clone Corp, Wuhan, China), Dll4 (10 ug/ml, PA5-46974, Thermo Fisher, Massachusetts, USA), Fn1 (1:200, 250073, Zenbio, China), and Notch3(1 ug/ml, ab23426, Abcam, Cambridge, UK). Tissue was fixed with paraformaldehyde and permeabilized with 0.3% Triton X-100 in PBS for 30 min. Tissue was blocked with blocking buffer (P0102, Beyotime, China) for 1 h at room temperature, followed by incubation with the primary antibody overnight at 4 °C. After incubation with secondary antibodies at a 1/500 dilution for 2 h, the sections were sealed with an antifade mounting medium with DAPI (P0131–25 ml, Beyotime, Shanghai, China). Images were acquired using a Zeiss AX10 microscope (Carl Zeiss, Weimar, Germany).

### Cell culture

The MC line SV40-MES-13(a mouse mesangial cell line, ATCC, Peking, China) was cultured in Dulbecco’s Modified Eagle Medium F12 (DMEM-F12, Gibco, Grand Island, NY, USA) supplemented with 10% fetal bovine serum (FBS) (Gibco, Grand Island, NY, USA) and 1% penicillin–streptomycin (Life Technologies, Carlsbad, CA, USA). The HUVECs (human umbilical vein endothelial cells, Qida, Shanghai, China) were cultured in ECM (1001, ScienCell, USA) under 5% CO_2_ at 37 °C.

### ELISA

To investigate the secretion of Dll4 by ECs, cell supernatant with or without LPS treatment (20 ug/ml, S1732, Beyotime, Shanghai, China) (26) was collected for ELISA. In clinical validation, human blood samples were centrifuged at 1,000 rpm to obtain the serum. The concentration of anti-Dll4 IgG in cell supernatant and serum samples was measured using ELISA kits (CSB-EL006949HU, CUSABIO, China). The assay were performed according to the manufacturer’s instructions. At the end of the incubation, stop solution was added and absorbance was measured at 450 nm by Microplate reader (Bio-Tek Corporation, California, USA).

### CCK8 assay

MCs (3×10^3^/well) were seeded in a 96-well plate, and 100 μl medium (DMEM-F12 with 10% FBS and 1% PS) was added to each well. After complete attachment, Dll4 (recombinant human Dll4, 100 ng/ml, HEK293 FC, MCE, USA) and Tarextumab (humanized anti-Notch2/3 antibody, 10 ug/ml, OMP 59R5, MCE, USA) were added to MCs. Tarextumab has been validated to bind and block mouse Notch3 with high affinity in previous reports (32). After 24 h incubation, cells were incubated in a 100-μl reaction mixture (10 μl CCK-8 and 90 μl DMEM-F12)(CCK-8, Beyotime, Shanghai, China) for 2 h and the absorbance of each well was measured at a wavelength of 450 nm by Microplate reader.

### RNA extraction and real-time reverse-transcription PCR

Total RNA was extracted using TRIZOL reagent (Life Technologies, Carlsbad, CA, USA) and was reverse transcribed to obtain cDNA using an RT-PCR kit (Promega, Madison, WISC, USA). qPCR was performed using an SYBR Green PCR Kit (Qiagen, Dusseldorf, Germany) in a StepOne Real-time PCR System (Applied Biosystems, Foster City, CA, USA). Fold differences were then calculated for each treatment group after ΔΔCt correction. The primers used were listed in Supplemental **Supplementary Table 3**.

### Western blot analysis

Cells were lysed on ice using RIPA lysis buffer supplemented with 1% protease inhibitor and 1% phosphatase inhibitor. The lysates were centrifuged at 12,000 × g and 4 °C for 15 mins, and the supernatants were collected for protein concentration determination via the BCA assay. Equal amounts of protein (20 µg per lane) were separated by 10% SDS-PAGE and transferred onto PVDF membranes. The membranes were blocked with 5% skim milk at room temperature for 1 h, followed by overnight incubation with primary antibodies at 4 °C. Primary antibodies used included: Notch3 (CST, 5276S, 1:1000), Hey1 (Proteintech, 19929-1-AP, 1:1000), Hey2 (Proteintech, 105791-1-AP, 1:1000), and β-tubulin (CST, #2128S, 1:1000). After washing with TBST, the membranes were incubated with HRP-conjugated secondary antibodies for 1 h at room temperature. Protein bands were visualized using an ECL reagent, imaged, and analyzed with ImageJ software to calculate the optical density ratio of target proteins relative to β-tubulin.

### Wound healing assay

Chambers were pasted in a 6-well plate and cells (1 × 10^4^/well) were seeded in chambers. After all cells had adhered to the walls and grown to confluence, chambers were pulled out vertically. The chambers were then rinsed twice with PBS, and a complete medium containing the Dll4 or Dll4 with Tarextumab was added. Images of the cells were captured using a microscope at 0, 6, 12, and 18 h. Wound closure was calculated using the following formula: (initial wound size-current wound size)/initial wound size×100 (33).

### siRNA knockdown of Dll4 in HUVECs

Dll4 siRNAs as listed in Supplemental **Supplementary Table 4**, were purchased from Gene Pharma, Shanghai, China. Cells were cultured to a density of 60%-70% before adding si-Dll4-RNA and transfected according to the manufacturer’s instructions of Gene Pharma.

### Transwell assay

In this study, a Transwell chamber system was employed to establish an indirect co−culture model of ECs and MCs. The experiment included three groups: control group, siRNA−Dll4 treatment group, and Tarextumab treatment group. LPS−pretreated ECs were seeded in 24−well plates at a density of 4×10^5^/ml (500 μl per well) and allowed to adhere overnight. Subsequently, MCs were seeded into Transwell inserts with an 8 μm pore size at a density of 2×10^5^/ml (200 μl per insert). After applying the respective treatments to each group, the Transwell inserts were transferred into the wells pre−seeded with ECs for 24−h co−culture. For migration detection, the upper chambers were filled with serum−free DMEM−F12 medium (100 μl per insert), while the lower chambers contained medium supplemented with 10% FBS (500 μl per well). Following incubation at 37 °C for 24 h, the inserts were removed, fixed with 4% paraformaldehyde, stained with 0.1% crystal violet, and examined under a microscope to count the MCs that had migrated to the lower surface of the membrane.

### EdU incorporation assay

In this experiment, three groups were established: control, siRNA-Dll4 treatment, and Tarextumab treatment groups. First, MCs were seeded overnight in a 24-well plate at a density of 4×10^5^/ml (500 µl per well). Subsequently, both si-Dll4-treated and untreated ECs (2×10^5^/ml, 100 µl per chamber) were seeded into 0.4 µm pore-sized Transwell inserts and co-cultured with the pre-plated MCs for 24 h, with Tarextumab added to one group of untreated ECs during co-culture. After co-culture, MCs were collected and re-seeded in a 24-well plate with 4% FBS at a density of 5×10^4^ cells per well. Following the BeyoClick™ EdU-594 kit protocol, 1 µM EdU was added to each well and incubated at 37 °C for 2 h. Cells were fixed with 4% paraformaldehyde for 30 minutes, permeabilized with 0.5% Triton X-100 for 10 minutes, stained using the Apollo reaction for 30 minutes, and counterstained with Hoechst for 30 minutes. EdU-positive nuclei were finally visualized and quantified under a fluorescence microscope.

### Statistical analysis

All experiments were repeated at least three times. Image analysis was performed using the ImageJ software. SPSS v24.0 (IBM, SPSS, Chicago, IL, USA) and GraphPad Prism 9 (GraphPad Software, La Jolla, CA, USA) was used for statistical analyses. Differences between two groups were analyzed using Student’s t-test, and multi-group comparisons were analyzed using one-way ANOVA. ROC curves were used for sensitivity and specificity analyses. P-value of <0.05 was considered statistically significant: *p < 0.05, **p < 0.01, and ***p < 0.001.

## Results

### Subclustering of ECs in the kidney of LN mice

**Figure 1:**
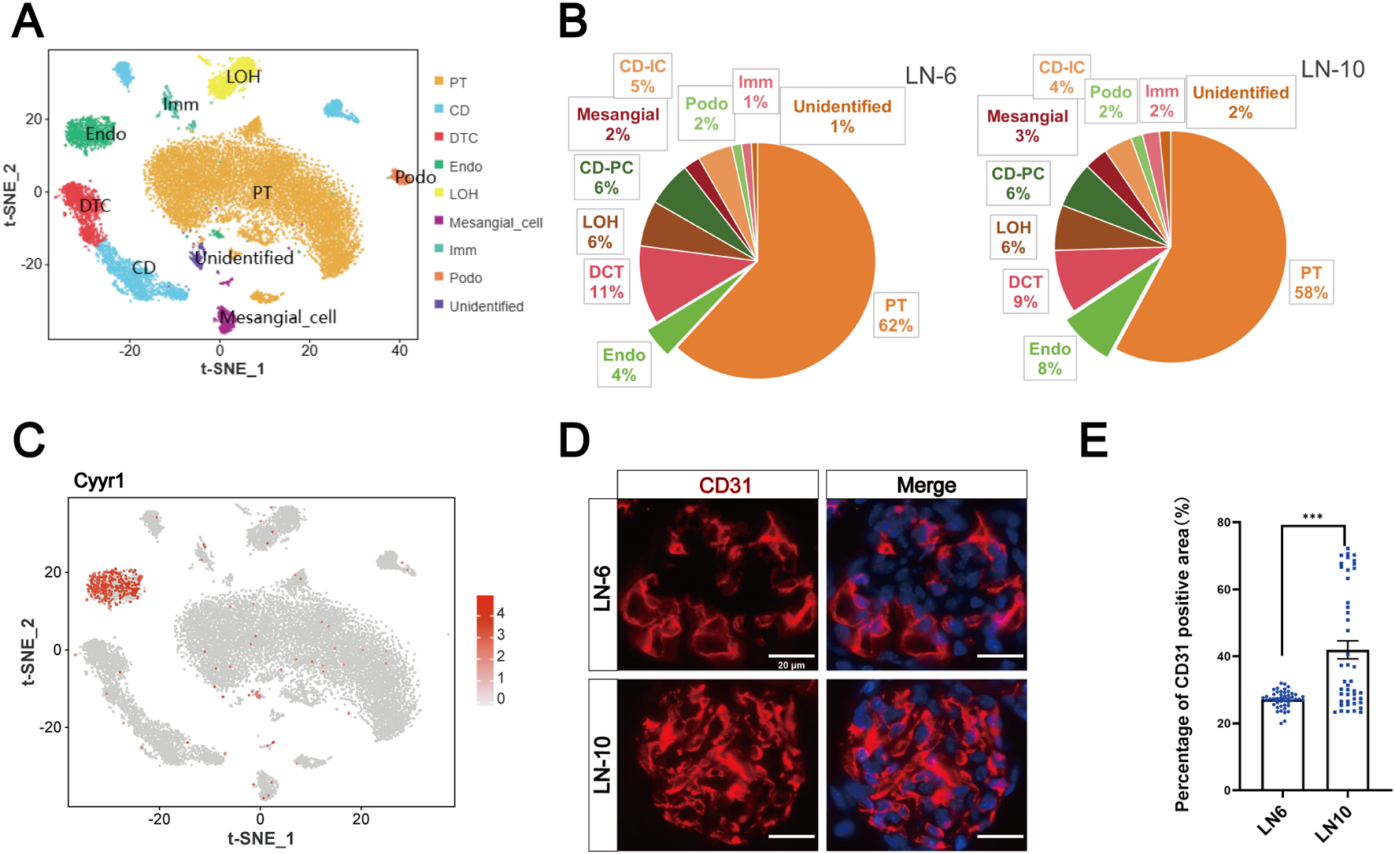
Heterogeneity of ECs in the kidneys of LN mice. **(A)** t-SNE analysis (left) showed nine different cell clusters in the kidneys of LN mice. Different cell clusters were color-coded. **(B)** Changes in the proportion of each cell cluster from 6 to 10 weeks in LN mice. **(C)** Expression distribution of marker gene in renal ECs of LN mice. **(D)** Immunofluorescence staining of CD31 (red) and nuclei (blue) in the kidneys of LN mice. Scale bar = 20 μm. **(E)** Percentage of CD31 positive area in the 6-week group in the renal section, randomly select 15 glomeruli from each group (n = 3), ***P < 0.001.

**Figure 2:**
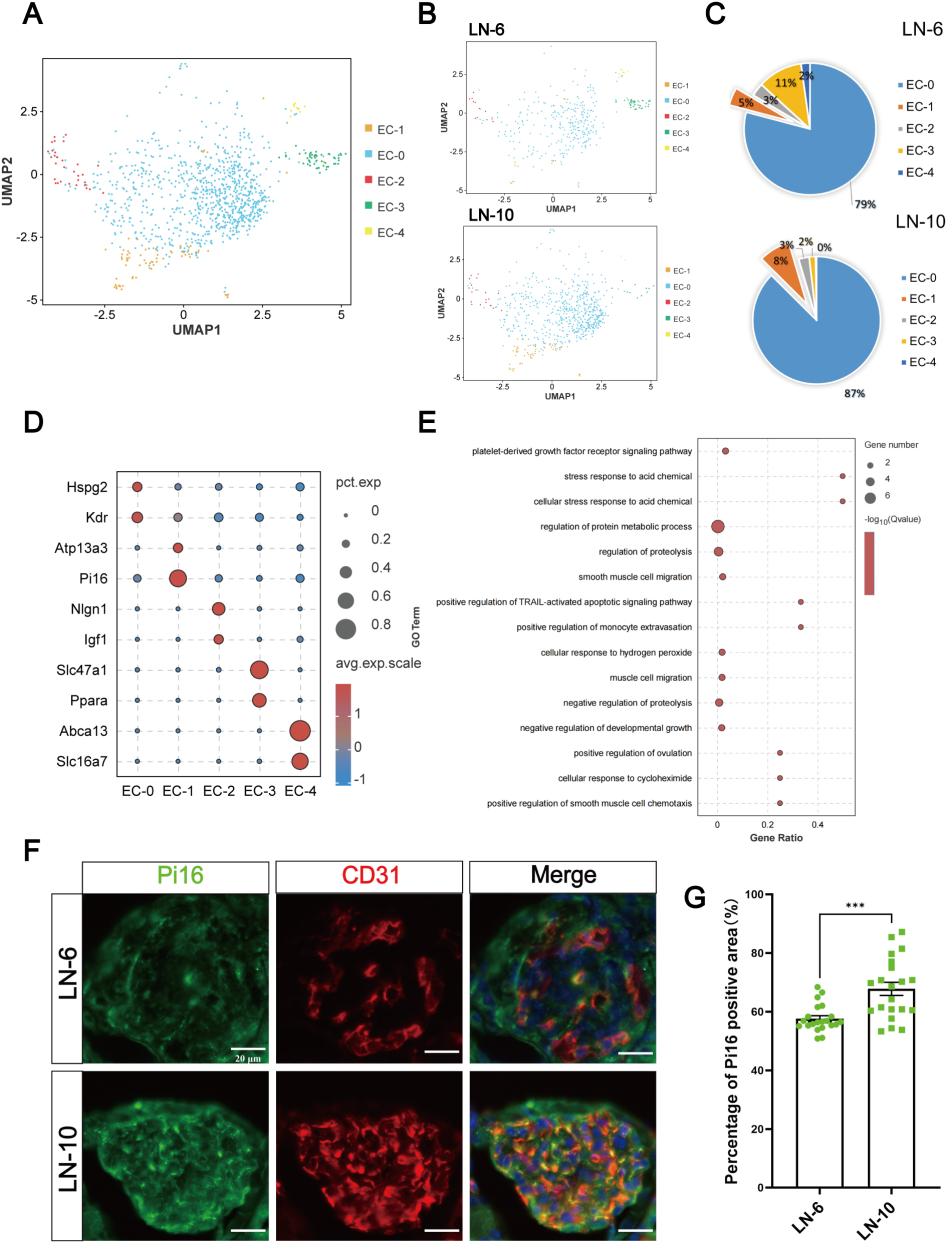
Subtype heterogeneity of ECs. **(A)** t-SNE analysis showing five EC subclusters. **(B)** t-SNE analysis showing the distribution of EC subtypes at 6 and 10 weeks. **(C)** Changes in the number of cells in EC subtypes in both the 6- and 10-week samples. **(D)** Bubble plots showing the expression of the top two marker genes in EC subclusters, with red color indicating higher expression. **(E)** Top 15 enriched GO BP terms of DEGs in EC-1. **(F)** Immunofluorescence staining of Pi16 (green) and CD31 (red) in the renal tissue sections. The yellow region is co-stained. Scale bar = 20 μm. **(G)** Analysis percentage of Pi16 positive signal area in EC-1 cells from 6-week and 10-week LN models, randomly select 7 glomeruli from each group (n = 3), (***p < 0.001).

According to the single-cell data of mouse glomerular canonical markers, we clustered cells in the kidney of LN mice into nine clusters (**Figure 1A**). Among them, the number of ECs was elevated in LN mice from 6 to 10 weeks of age, consistent with the findings of the current study (**Figure 1B**). t-SNE map showed the expression distribution of EC marker gene Cyyr1 (**Figure 1C**). CD31 immunofluorescence staining was used to evaluate the changes in the expression of renal ECs in LN mice at 6 W and 10 W, indicating that the number of renal vascular ECs in LN mice increased significantly with disease progression (**Figures 1D, E**). Therefore, we performed an unbiased cluster analysis of 8398 ECs and identified five subclusters of ECs (EC 0-4) (**Figure 2A**). From 6 to 10 weeks, the proportions of EC-0 and EC-1 increased, with more significant increases in the EC-1 clusters (**Figures 2B, C**). Marker gene bubble maps were constructed for the five EC subclusters (**Figure 2D**). The top 5 marker genes for each subcluster are listed in **Supplementary Table 5**. Gene Ontology (GO) enrichment analysis for biological processes (BP) was performed on the upregulated genes from the EC-1 cluster (**Figure 2E**). And the other clusters were shown in **Supplementary Figure 1**. EC-1 was mainly involved in cell regulation of protein metabolic process. Next, we performed immunofluorescence co-staining of the EC-1 marker gene Pi16 and the EC marker gene CD31 in kidney sections of LN mice at 6 and 10 weeks. The results demonstrated that the number of EC-1 cells significantly increased with disease progression, which coincided with the previous snRNA-seq analyses (**Figures 2F, G**).

### The communication network of EC subclusters in LN

**Figure 3:**
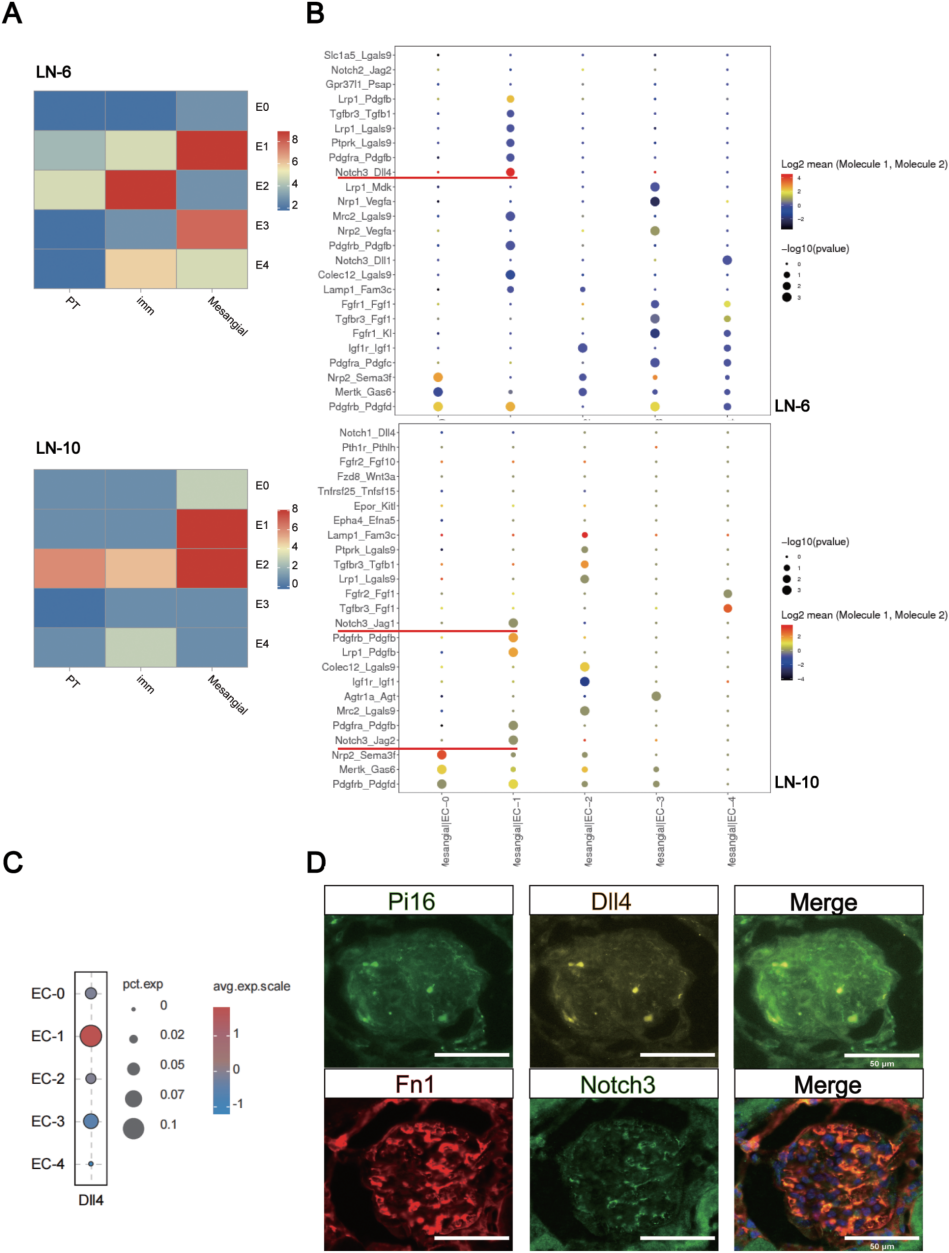
Interaction network between ECs and other cell types in the kidneys of LN mice. **(A)** Heatmap of cell expression correlation between EC subtypes and other cell types. The color denotes the correlation coefficients between the ligand-receptor pair and the cell types. **(B)** Ligand-receptor relationship between MCs and EC subclusters, when MCs were the receptor cells. **(C)** Bubble plot showing the expression of Dll4 in EC subclusters. **(D)** Co-immunolocalization of Dll4 and Pi16 on EC-1, and co-immunolocalization of Notch3 and Fn1 on MCs. Scale bar = 50 μm, n = 3.

As shown in **Figures 3A**, there was a strong reciprocal relationship between MC and EC-1. **Figure 3B** further suggests the ligand-receptor interactions between MC and EC-1 when MC functioned as receptors. Bubble plots showed the expression of Dll4 in the EC subclusters (**Figure 3C**). The results were further confirmed by immunofluorescence staining of Pi16 (marker gene of EC-1) and Dll4 in the kidney tissue of LN mice (**Figure 3D**). In addition, we performed co-localization staining of Fn1 (a classical marker for MC) and Notch3 in the kidneys of LN mice and verified the expression of Notch3 in the MC (**Figure 3D**). The result showed that EC-1 might secrete Dll4 to interact with the receptor Notch3 in MCs.

### Dll4 promotes the proliferation and migration of MCs via Notch3 *in vitro*

**Figure 4:**
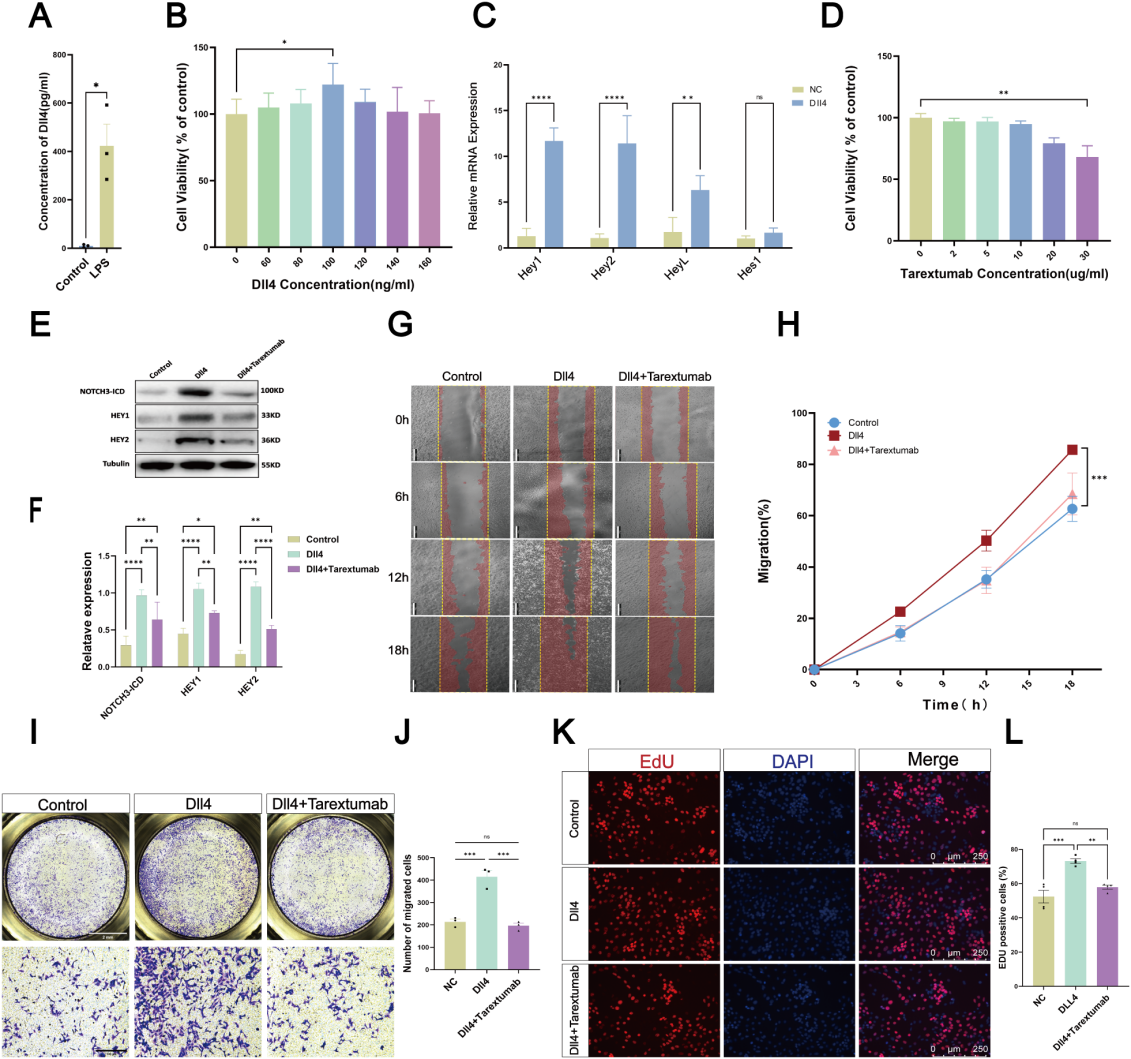
EC regulate MC via the Dll4/Notch3 axis. **(A)** ELISA assays were performed to detect the expression of Dll4 in ECs after LPS stimulation. **(B)** CCK8 assay showing the effects of Dll4 on MC viability. **(C)** qRT-PCR assay showing Notch3 relative genes were upregulated in MC after Dll4 stimulation. **(D)** CCK8 assay showing the effects of Tarextumab on MC viability after Dll4 interference. **(E)** Western blotting analysis of the expression of the Notch3-ICD and its downstream-related proteins HEY1 and HEY2 following interference with Dll4 alone or in combination with Tarextumab. **(F)** Quantification of Western blotting. Data were representative of three independent experiments (n = 3 per group). **(G)** Representative images of wound healing assays showing the migration of MC cells treated with Dll4 or Dll4 in combination with Tarextumab. Scale bar = 200 μm. **(H)** Quantitative comparison of MC cell migration rates among Control, Dll4, and Dll4+Tarextumab treatment groups (n = 3). **(I)** Transwell analysis of the effect of Dll4 and Tarextumab on MC cell migration. Scale bar = 2 mm for overview. Scale bar = 300 μm for magnified view. **(J)** Quantitative analysis of the number of migrated cells. Data are presented as mean ± SD (n = 3). *p < 0.05, **p < 0.01. **(K)** EdU assays were performed to detect the proliferation of MC treated with Dll4 and Tarextumab. Scale bar = 200 μm. **(L)** Quantitative analysis of MC cell proliferation by EdU assay. Data are presented as mean ± SD (n = 4). *P < 0.05, **P < 0.01, ***P < 0.001, ****P < 0.0001.

*In vitro*, we used LPS, a specific potent pro-inflammatory cytokine, to activate ECs and detect Dll4 expression by ELISA test (**Figure 4A**). Also we performed qRT-PCR to detect the mRNA expression change of Dll4 in ECs after TNF-α and LPS stimulation (**Supplementary Figure 2**). The results showed that the expression of Dll4 was increased. After treating MCs with Dll4, CCK8 testing was performed to verify cell viability, as shown in **Figure 4B**. The results showed the 100 ng/ml was the optimal viability concentration. We then chose 100 ng/ml Dll4 to activate MCs and detected Notch3 target genes (*Hey1, Hey2, HeyL, Hes1*) *(*34, 35) by qRT-PCR. (**Figure 4C**). The results showed that Dll4 could promote the mRNA expression of Notch3 downstream genes in MCs. To further validate the activation of the Notch3 signaling pathway in MCs upon Dll4 stimulation, we performed Western blot analysis to detect the expression of the Notch3 intracellular domain (NOTCH3-ICD) and its downstream targets Hey1 and Hey2. Compared with the control group, stimulation with recombinant Dll4 for 48 h significantly increased the protein levels of cleaved NOTCH3-ICD, Hey1, and Hey2 in MCs. In addition, we also added a Notch2/3 inhibitor Tarextumab with Dll4. After CCK8 testing, as shown in **Figures 4D**, we chose a concentration of 10 µg/ml of Tarextumab for further analyses. The results showed that the addition of Tarextumab with Dll4 effectively attenuated Dll4 induced upregulation of Notch3 pathway (**Figures 4E, F**). To explore the effect of the Dll4/Notch3 axis on MC migration capacity, we performed Wound healing and Transwell assays. Dll4 was shown to promote MC migration compared with that in the control group. In contrast, the migration ability was notably attenuated after adding Tarextumab to the culture medium (**Figures 4G–J**). To detect the effects on MC proliferation capacity, we performed an EdU assay. The percentage of EdU-labeled proliferating cells increased significantly after the addition of Dll4, whereas Tarextumab restored the proliferation induced by Dll4 (**Figures 4K, L**). These results indicate that Dll4 regulates the migration and proliferation of MCs through Notch3.

### Interference of the Dll4/Notch3 axis inhibits MC migration and proliferation

**Figure 5:**
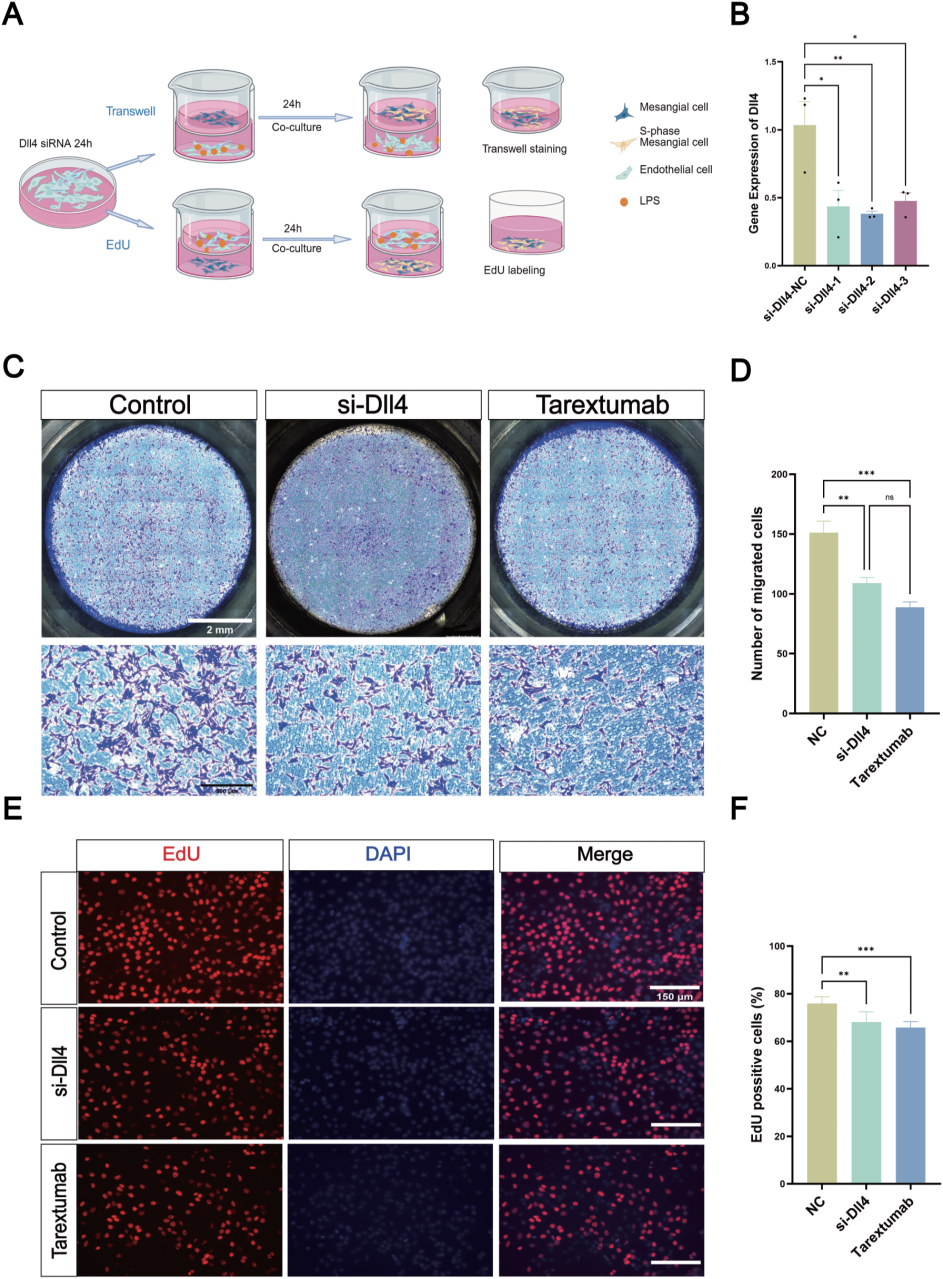
Dll4 promotes the proliferation and migration of MC via Dll4 *in vitro*. **(A)** Co-culture illustration of ECs and MCs according to different treatment, the control group was treated with LPS to ECs alone, siRNA-Dll4 group was siRNA-Dll4 in ECs then treated with LPS, Tarextumab group was ECs treated with LPS and MCs treated with Tarextumab. **(B)** qRT-PCR results of the Dll4 mRNA expression after Dll4 siRNA transfection in ECs (n = 3, *P < 0.05, **P < 0.01). **(C)** Transwell assay showing the migration of MC in different groups, Scale bar = 2 mm (upper), Scale bar = 300 μm (lower). **(D)** MC migration statistics (n = 3, **P < 0.01, ***P < 0.001). **(E)** EdU assays were performed to characterize the proliferation of MC when knocking down Dll4 in ECs or adding Tarextumab. Scale bar = 150 μm. **(F)** MC proliferation statistics n = 3 for each group, **P < 0.01, ***P < 0.001.

**Figure 5** A showed the schematic diagram of the co-culture system of ECs and MCs. We first knocked down Dll4 in ECs, and the results showed that siRNA-Dll4–2 exhibited the highest silencing efficiency (**Figure 5B**). We then performed Transwell and EdU assays on MCs to detect the effects of Dll4 siRNA on the migration and proliferation of MCs. Interfering with Dll4 in ECs or adding Tarextumab in MCs before LPS treatment on ECs can decrease co-cultured MC migration significantly compared to the control group, especially in the Tarextumab group (**Figures 5C, D**). The EdU assay results showed that knockdown of Dll4 in ECs significantly inhibited MC proliferation, and the interference of MC with Tarextumab treatment further reduced MC proliferation (**Figures 5E, F**). These data are consistent with the hypothesis that EC might regulate glomerular MC migration and proliferation through the Dll4/Notch3 axis.

### Evaluation of Dll4 as a candidate predictor of disease activity in patients with non-renal SLE and LN

**Figure 6:**
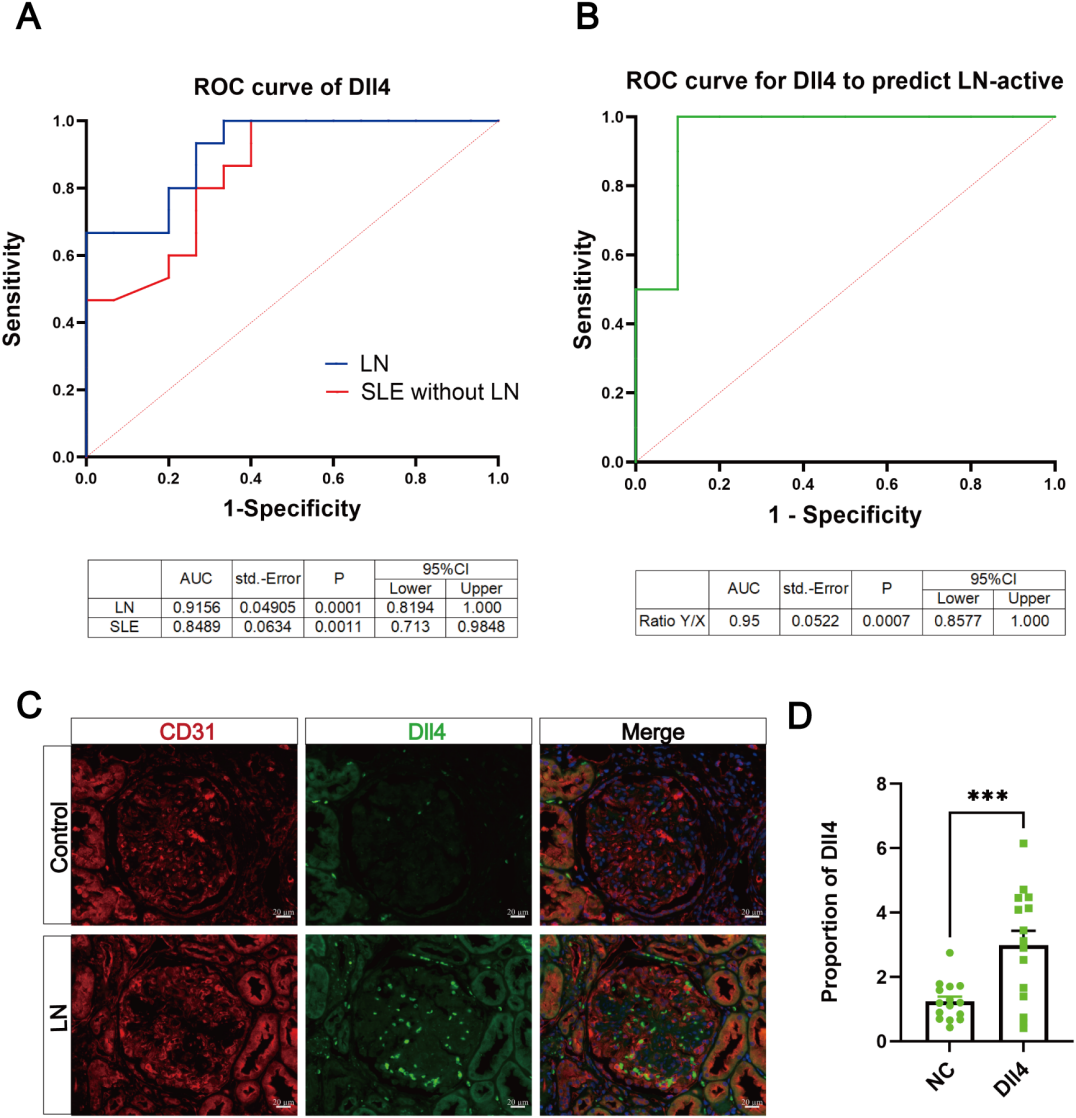
Evaluation of Dll4 as a predictor of disease activity in patients with non-renal SLE and LN. **(A)** ROC curves of the sensitivity and specificity of Dll4 in patients with non-renal SLE and LN. **(B)** ROC curve of the sensitivity and specificity of Dll4 in patients with active LN. **(C, D)** Dll4 immunofluorescence staining of renal sections in the control group (healthy normal kidneys of renal cancer patients) and LN patients. Scale Bar = 20 μm, n = 3, ***P <0.001.

As shown in **Figures 6A**, ROC curve analysis revealed that serum Dll4 levels were significantly elevated in both non-renal SLE and LN patients compared to the healthy control group. **Figure 6B** further demonstrated that serum Dll4 levels were markedly increased in patients with active LN relative to those in the inactive disease phase. Renal sections of LN patients were subjected to immunofluorescence staining for CD31 and Dll4. The results showed that the expression of Dll4 in renal vascular ECs of LN patients was significantly greater than that in the relatively healthy normal group (**Figures 6C, D**). These findings further support a correlation between Dll4 and disease activity in LN patients.

## Discussion

SLE remains a challenging autoimmune disease that is difficult to diagnose and treat. The lack of targeted therapies, combined with the heterogeneity of disease manifestations and treatment responses, highlights the necessity of revealing the molecular and cellular networks of this disease (36). snRNA-seq has multiple advantages, including an unbiased genomewide quantification of molecules in thousands of individual cells, as well as multiplex spatial analysis of proteins and RNA in situ (37), elimination of dissociation-induced transcriptional stress responses (38–40), and provided a resource for identifying novel disease-related genes and pathways (41). In this study, we used snRNA-seq to identify the heterogeneity and subclusters of ECs in the kidneys of LN mice. To delineate changes associated with disease progression, we compared MRL/Lpr mice at an early stage (6 weeks) with those at a later stage of established nephritis (10 weeks). We then focused on the molecular interactions between ECs and MCs, and identified that EC-1 might regulate the proliferation and migration of glomerular MCs via the Dll4/Notch3 axis in LN.

The kidney is a highly vascularized organ. ECs in the microvasculature regulate blood flow and modulate coagulation, inflammation and vascular permeability (42), playing an important role in maintaining renal function. The number of ECs increased in LN mice from 6 to 10 weeks (16), and the pathological alterations of glomerular ECs in LN are evident (43). During glomerular injury, circulating pro-inflammatory cytokines and the deposition of immune complexes contribute to endothelial dysfunction (43). Inflammation related pathways are enriched in ECs after acute kidney injury, emphasizing the pro-inflammatory and anti-angiogenic state of the damaged endothelium (44). The endothelial damages then drive further kidney injury and systemic complications. In addition, recent studies have revealed that vascular ECs are heterogeneous in different tissues and that this heterogeneity plays a key role in a variety of disease models (23, 24).

MCs are stromal cells that are important for glomerular homeostasis and the glomerular response to injury (13). In LN, MCs constantly undergo severe damage, resulting in excessive proliferation and increased extracellular matrix production (45, 46). Here, we focused on the EC heterogeneity in LN and investigated the regulatory role of ECs on MCs. It was discovered that in LN, EC-1 might regulate the migration and proliferation of glomerular MCs through the Dll4/Notch3 axis.

Dll4 is a Notch ligand primarily expressed in ECs and is crucial for the activation of the Notch signaling pathway (47–49). Previous studies have highlighted the relevance of Notch signaling cascade in the fibrogenic response (50). Notch3 activation has been observed in glomeruli from rats with mesangial proliferative nephritis (51). These findings strengthen the evidence that the Dll4/Notch3 axis is functionally active in MCs and contributes to their pathological behavior in LN. Notch expression is increased in the kidneys of LN patients and is important in the development of renal fibrosis, suggesting that Notch inhibition may limit kidney destruction (47). Some studies have shown that inhibitors of Notch Signaling are an effective treatment for autoimmune (51).

Here, we found Tarextumab, a novel cross-reactive antibody that binds to and selectively inhibits Notch2/3 (52, 53), could reduce the effect of Dll4 on MCs, suggesting that ECs might secret Dll4 to regulate MCs through Notch3 pathway. Therefore, the Dll4/Notch3 axis emerges as a candidate strategy worthy of further investigation for treating LN. This study also has certain limitations. In the *in vitro* experiments, LPS was used to activate ECs, yet this stimulus is not a specific pathogenic factor in LN. Future studies should apply stimuli more closely aligned with the pathophysiological features of LN, such as immune complexes or type I interferons, to further validate the biological significance of the Dll4/Notch3 axis. Moreover, the current findings are primarily based on *in vitro* experiments and lack direct *in vivo* evidence linking this pathway to the pathogenesis of LN. In subsequent work, we plan to utilize lupus mouse models for *in vivo* functional validation to clarify the causal role of Dll4/Notch3 axis and its functional contribution to LN progression.

**Figure 7:**
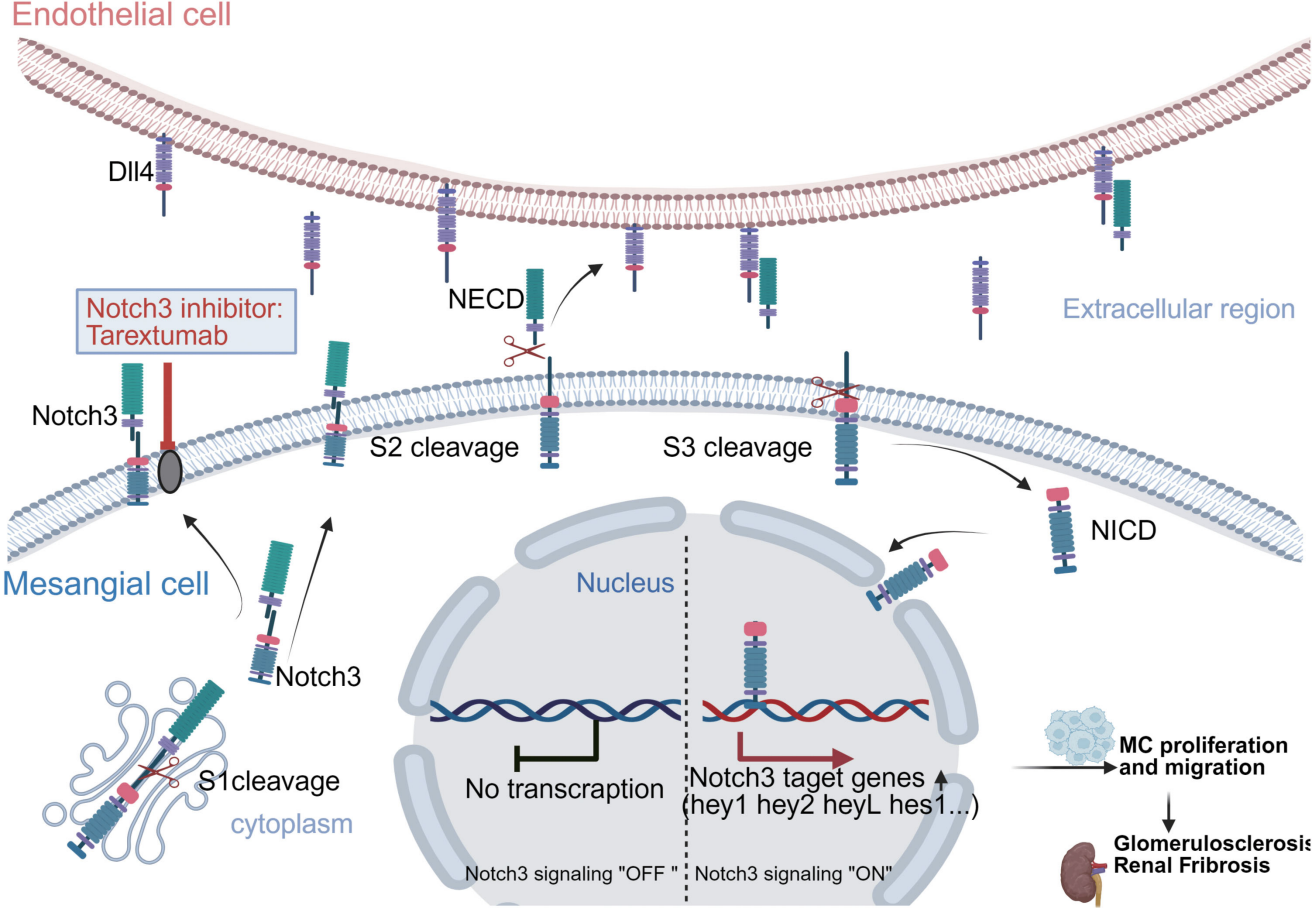
Schematic illustrating the Dll4-Notch3 axis between EC and MC in the glomeruli of LN. In the early stages of LN, EC regulate MC through the Dll4/Notch3 axis to initiate pathogenesis and affect MC proliferation and migration. As the disease progresses, the proliferation and migration of MC damage the structure and function of the glomerulus, aggravating renal injury and leading to glomerulosclerosis and renal fibrosis.

In conclusion, by integrating snRNA-seq analyses with preclinical validation, we have defined EC heterogeneity in LN and uncovered a potential signaling axis between ECs and MCs via Dll4/Notch3. Our *in vitro* findings demonstrate that intervention of the Dll4/Notch3 axis can modulate MC phenotypes. Collectively, our work suggests that the Dll4/Notch3 axis might be a promising candidate pathway for future therapeutic development in LN (**Figure 7**).

## Data Availability

The raw data supporting the conclusions of this article will be made available by the authors, without undue reservation.
