# Correction: Endothelial cells regulate mesangial cells through the Dll4/Notch3 axis to participate in glomerular injury in lupus nephritis

**DOI:** 10.3389/fimmu.2026.1832579

**Published:** 2026-03-26

**Authors:** Haixia Guo, Yueying Mao, Yongjun Wang, Bin Yu, Zhifeng Gu, Chun Yao, Zhanyun Da

**Affiliations:** 1 Department of Rheumatology, Affiliated Hospital of Nantong University, Medical School of Nantong University, Nantong, Jiangsu, China; 2 Department of Endocrinology and Rheumatology, Tinghu District People’s Hospital, Yancheng, Jiangsu, China; 3 Key Laboratory of Neuroregeneration of Jiangsu and Ministry of Education, Co-innovation Center of Neuroregeneration, Nantong University, Nantong, Jiangsu, China

**Keywords:** Dll4/Notch3, endothelial cells, lupus nephritis, mesangial cells, tarextumab

The funder “Research Discipline Development Foundation of Affiliated Hospital of Nantong University, YJXYY202204-XKB03” and the funder “Nantong Natural Science Foundation, JC:2025074” were erroneously omitted. The corrected **Funding** statement has been updated to:

“The author(s) declared that financial support was received for this work and/or its publication. Research Discipline Development Foundation of Affiliated Hospital of Nantong University (YJXYY202204-XKB03) and Nantong Natural Science Foundation (JC:2025074).”

The original version of this article has been updated.

