# Supplementary material for: Endothelial cells regulate mesangial cells through the Dll4/Notch3 axis to participate in glomerular injury in lupus nephritis
Source: Front Immunol. 2026 Mar 13;17:1720756. doi: 10.3389/fimmu.2026.1720756 (PMC13062793; doi:10.3389/fimmu.2026.1720756)
Supplement: Supplementary file 1 [file Table1.docx]

**Supplementary Figures**


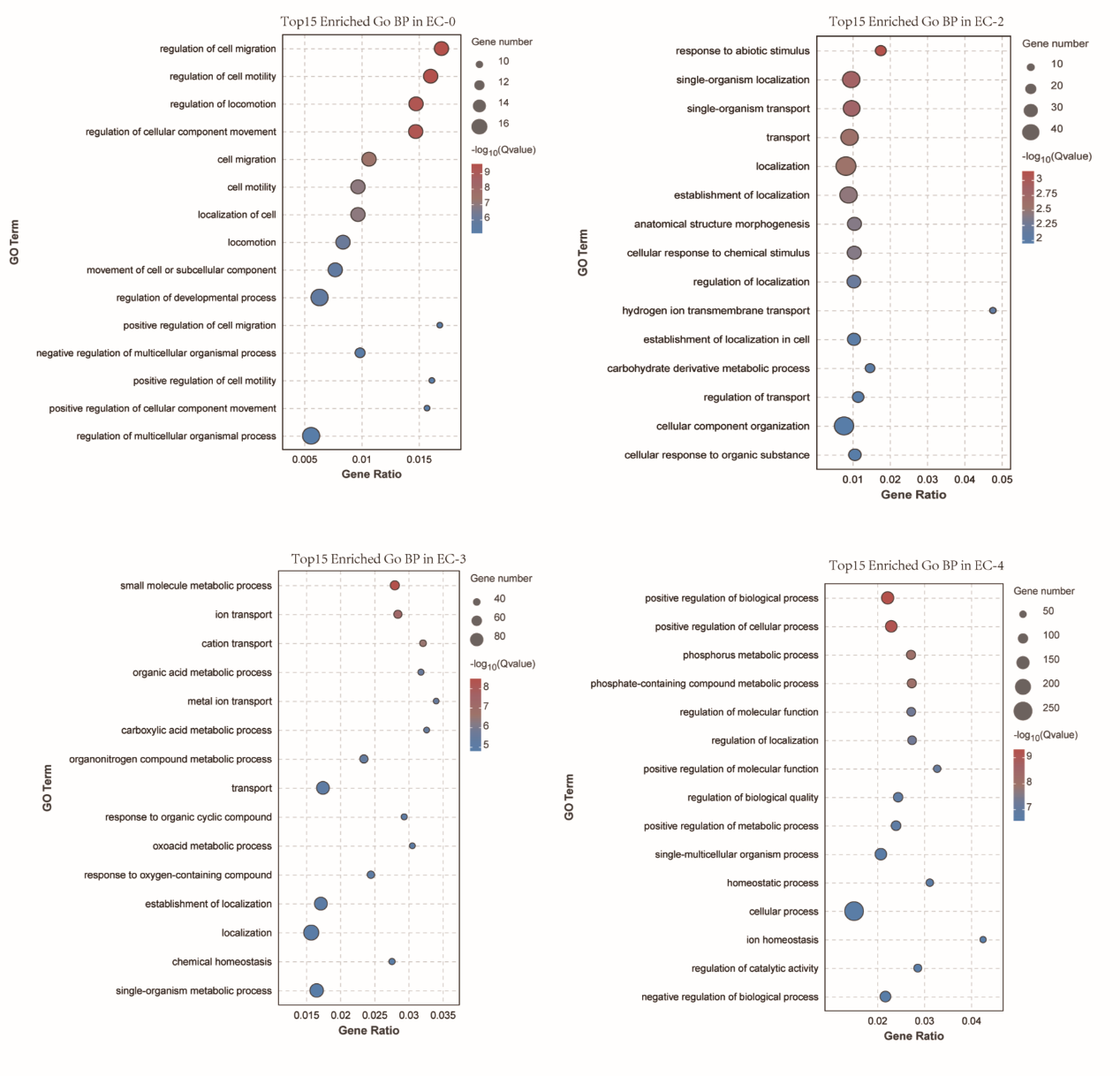


**Figure S1**. The Gene Ontology (GO) enrichment of the biological process (BP) category top 15 among the upregulated genes in the EC-0, EC-2, EC-3 and EC-4.


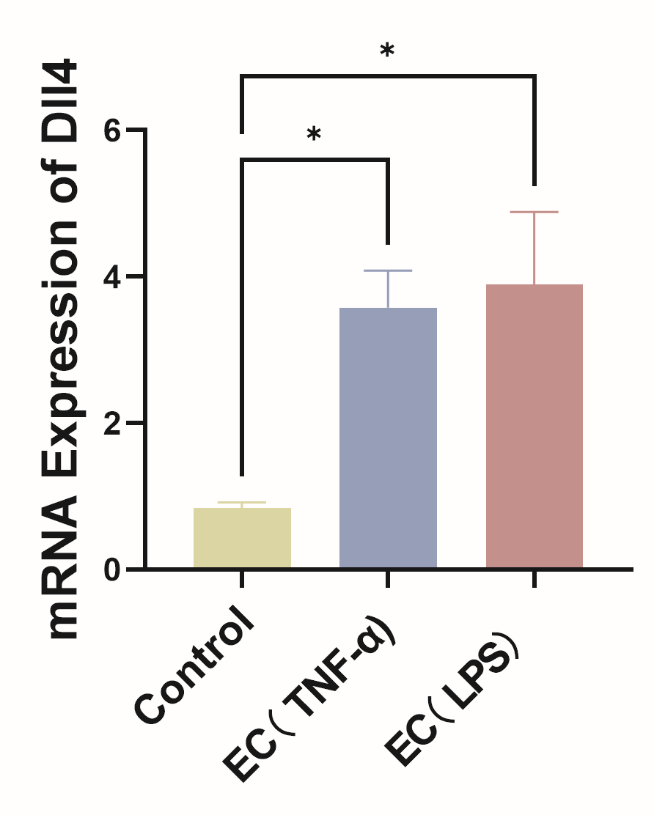


**Figure S2**. qRT-PCR to detect the mRNA expression change of Dll4 in ECs after TNF-α and LPS stimulation.
