# Supplementary material for: Endothelial cells regulate mesangial cells through the Dll4/Notch3 axis to participate in glomerular injury in lupus nephritis
Source: Front Immunol. 2026 Mar 13;17:1720756. doi: 10.3389/fimmu.2026.1720756 (PMC13062793; doi:10.3389/fimmu.2026.1720756)
Supplement: Supplementary file 2 [file Table2.docx]

**Supplementary Tables**

Table S1. Demographic and clinical characteristics of the participants with SLE and LN and Healthy people.

Table S2. Demographic and clinical characteristics of the participants with active LN and low active LN.

Table S3. Sequences of Primers for qRT-PCR.

| **Gene** | **Sense** | **Antisense** |
| --- | --- | --- |
| *β-actin* | 5’-GAAGATCAAGATCATTGCTCCT-3’ | 5’-TACTCCTGCTTGCTGATCCA-3’ |
| *Dll4* | 5’-TACACCGACCTCTCCACAGACACCTT-3’ | 5’-AGTCCGACAAGTTGTTCATGGCTTCC-3’ |
| *Hes1* | 5’-CAGCCAGTGTCAACAAGACACCGGACAAAC-3’ | 5’-TGCCCTTCGCCT-3’ |
| *Hey1* | 5’-AAAGACGGAGAGGCATCATCG-3’ | 5’-GCAGTGTGCAGCATTTTCAGG-3’ |
| *Hey2* | 5’-GTGGGGAGCGAGAACAATTA-3’ | 5’-GTTGTCGGTGAATTGGACCT-3 |
| *HeyL* | 5’-CTGAATTGCGACGATTGGT-3 | 5’-GCAAGACCTCAGCTTTCTCC-3 |

Table S4. Sequences for Dll4 si-RNA.

| **si-RNA** | **Sense** | **Antisense** |
| --- | --- | --- |
| Dll4-1 | 5’-GUGUCUGCCUUAAGCACUUTT-3’ | 5’-AAGUGCUUAAGGCAGACGCTT-3’ |
| Dll4-2 | 5’-GGCUACUAUGGCCUGCAUUTT-3’ | 5’-AAUGCAGGCCAUAGUAGCCTT-3’ |
| Dll4-3 | 5’-CCUGUGCAAGAAGCGCAAUTT-3’ | 5’-AUUGCGCUUCUUGCACAGGTT-3’ |

Table S5. The expression of the top 5 marker genes in EC subclusters.
